# The Individual Green-Washing Effect in E-Mobility: Emotional Evaluations of Electric and Gasoline Cars

**DOI:** 10.3389/fpsyg.2021.594844

**Published:** 2021-05-20

**Authors:** Petra Jansen, Franziska Anna Schroter, Philipp Hofmann, Ronja Rundberg

**Affiliations:** Faculty of Human Sciences, University of Regensburg, Regensburg, Germany

**Keywords:** E-mobility, sustainability, pro-environmental behavior, mindfulness, implicit and explicit attitude

## Abstract

In this study, the affective explicit and implicit attitudes toward electric and gasoline cars are investigated. One hundred sixty-five participants (103 cisgender women, 62 cisgender men) completed an explicit and implicit affective rating task toward pictures of electric and gasoline cars, measurements of sustainability, future and past behaviors, and mindfulness. The results showed a positive emotional attitude for the electric cars compared with the gasoline cars only for the explicit rating but not for the implicit one. Furthermore, factors that correlated to the attitudes were investigated: explicit ratings in car owners correlated with age, degree, sustainability in general, and the expressed intention to purchase an electric car in the future. Implicit attitudes in car owners correlated with the overall score of mindfulness and the dimension of “non-reactivity.” For the non-car owners, explicit attitudes correlated with the expressed intention to purchase an electric car in the future and the mindfulness dimension of “describing”. In this group, the implicit attitude correlated negatively with the mindfulness intention of acting with awareness. This indicates that several different factors should be considered in the development of promotion campaigns for the advantage of sustainable mobility behavior.

## Introduction

One reason for air pollution and climate change is vehicular emission (Sims et al., 2014). For example, while in 2000 around 800,000 people died from air pollution, this number rose to 3.2 million people in 2010. Behavioral, technical, and economic changes are necessary to decrease pollution. One important area of interest is sustainable transportation, which includes measures like the promotion of the public transportation system and the development of perfected e-mobility systems. Electric vehicles reduce carbon dioxide emissions and seem to be a promising sustainable transportation solution (Holdway et al., 2010).

To support the acceptance of promising ecological transportation solutions, it is important to investigate the relevant factors for sustainable behavior. A first study on sustainable transportation behavior has been conducted by Manca et al. (2019). They identified past sustainable behaviors, attitudes, and emotions as the key determinants of pro-environmental behavior: for the change in travel behavior, habit deactivation is a necessary but not a sufficient condition (Thøgersen and Møller, 2008). This gives a hint that attitudes and emotions do play an important role in behavioral change. While investigating the effect of argument quality on explicit and implicit attitudes of sustainable transportation, Manca et al. (2019) carved out the following picture: if the participants were highly involved in the topic of sustainable transportation, implicit attitudes were more positive in the condition where the arguments came from high source expertise (e.g., from a Nobel Prize winner), which is in contrast to the implicit attitudes of participants with low involvement. This study highlighted that the elaboration process for changing attitudes is driven by affective and cognitive aspects. Furthermore, the results of their study demonstrated that the fear to receive negative consequences of a potential unsustainable behavior might be the main predictor for the pro-environmental choice in the explicit rating. Both implicit and explicit attitudes were not correlated, but both are important to predict the behavioral intention. Their results confirm the Elaboration Likelihood Model (ELM, Petty and Cacioppo, 1996), which assumed two routes of persuasion, a central and peripheral route. While the central route demands more time for evaluating the message with the former existing schema, the peripheral route describes an attitude change, which might be less enduring.

Because the former consistent existing schemas are a key element in the change to pro-environmental behavior, it is important to investigate the underlying attitudes, which form cognitive schemata. This is valuable, because especially in environmental psychology the connection between attitudes and behaviors could be demonstrated (Crano and Prislin, 2006; Bamburg and Möser, 2007). Until now, research in psychology has focused, e.g., on the relevance of psychological correlates of pro-environmental attitudes and behaviors (Soutter et al., 2020). In a meta-analysis, it has been carved out that openness and honesty–humility demonstrate the strongest correlation of pro-environmental attitudes. The authors argue that it is wise to consider the roles of individual differences when investigating pro-environmental attitudes and behaviors.

One individual difference can be seen in the disposition of mindfulness. Mindfulness, which describes the ability to be completely, in a non-judgmental way, aware of the present moment (Kabat-Zinn, 1990), can play an important role in building up the necessity to change the own behavior toward sustainability. According to Hölzel et al. (2011), attention regulation, body awareness, emotion regulation, and change in perspective of the self are the mechanisms behind mindfulness. Different forms of meditations can be used as elements of mindfulness interventions as, for example, attentional, constructive, and deconstructive meditation practices (Dahl et al., 2015). A relation between mindfulness and sustainable behavior might be obvious: on the one side, mindfulness can support a sustainable lifestyle because it increases subjective well-being (Fischer et al., 2017); on the other side, it can increase human values and empathy toward other beings (Ericson et al., 2014). However, there seems to be a blind spot in the theoretical, conceptual, and empirical academic debates regarding the relevance of mindfulness in sustainability research (Wamsler et al., 2018).

For this, the relation of mindfulness and sustainable behavior has to be investigated carefully. This has been done in one study by Hunecke and Richter (2019) examining the connection of different aspects of mindfulness, construction of meaning, and sustainable food consumption. One result was that there was a relation between acting with awareness and sustainable food consumption. This relation was not visible if the dependent variable was the choice of a vegetarian lifestyle, providing evidence that vegetarianism is more explained by moral arguments. However, the study is limited by the fact that sustainable behavior, in this case food consumption, is retrieved from self-reports. In addition, some of the items of the Five Facet Mindfulness Questionnaire (FFMQ) were excluded to improve the fit of the model. The FFMQ (Baer et al., 2008; Michalak et al., 2016) is a dispositional mindfulness questionnaire that covers the subscales of observing (experience of inner and outer stimuli), non-reactivity (the ability not to react directly), acting with awareness (acting with full attention), non-judging (taking things as they are), and describing (inner labeling of things).

The main goal of the study presented here is to investigate the relation of mindfulness to the explicit and implicit attitudes toward e-cars. Explicit and implicit evaluations refer to the controlled (or conscious) and uncontrolled (or unconscious) aspects of human behavior, which can be explained by dual-process or dual-system models (Cameron et al., 2012). We will use an affective priming task, which focuses on the affective component of implicit evaluations, in contrast to the Implicit Association Test (IAT), which mainly reflects associations between concepts and the cognitive representation of attitudes toward a task (Brand and Ekkekakis, 2018). An affective priming effect can be explained as follows: the “spreading activation mechanism” suggests that targets with a congruent valence activate a certain response pathway, facilitating a quick response. In contrast, targets with an incongruent valence will initiate the wrong response pathway. To enable the correct response, the wrong pathway has to be inhibited first (Fazio et al., 1995). Investigating implicit, as well as explicit, attitudes might give a hint why consumers report favorable attitudes toward pro-environmental behavior but do not demonstrate sustainable action (White et al., 2019).

According to the literature presented above, the following hypotheses will be investigated:

1. First, due to the well-known social desirability bias (Chao and Lam, 2011) and the relevance of sustainability due to climate change, it is assumed that electric cars are explicitly evaluated more positively than gasoline cars. Consequently, we expect implicit attitudes to be independent of the explicit ones. Besides, in two further analyses, the factors “ownership of a car” and “gender” are included in the analyses. Because we expect that the implicit and explicit ratings are more positive if the topic is relevant for the participants, we assume a different attitude toward electric cars between car owners and non-car owners. Furthermore, we assume that women show a more positive attitude toward e-cars than men because women report greater pro-environmental views and concern about environmental problems (Xiao and McCright, 2015).
2. Second and according to Cameron et al. (2012) and Manca et al. (2019), we assume–if at all–that only a small correlation between explicit and implicit attitudes is expected.
3. Third and according to the study of Hunecke and Richter (2019), a correlation between acting with awareness and the explicit positive attitude of e-cars is expected. Because Lueke and Gibson (2015) demonstrated that mindfulness can reduce the automatic activation of negative implicit association (in their study toward older people), non-reactivity is assumed to correlate with implicit attitudes toward e-cars, too. In an exploratory analysis, it will be examined if mindfulness (measured with the FFMQ), sustainability [according to von Behren et al. (2018), Hunecke and Richter (2019), and Manca et al. (2019)] demographic variables (e.g., age and degree), and future and past behaviors predict the explicit and implicit attitudes toward electric cars. Besides, analyses will be calculated separately for people who own and those who do not own a car.

## Materials and Methods

### Participants

Two hundred and ten participants completed the experiment. They were recruited *via* the newsletter of the faculty of human science of the university and social media. Students received course credit for their participation. Forty-five had to be excluded because they had more than 10% missing values in the implicit or explicit tasks or more than 50% error trials in the implicit task, resulting in 165 participants in the final sample. For the gender item, the option “other” was offered, but it was not chosen by any participant. None of the participants owned an e-car.

With a medium effect size of *f* = 0.25, an alpha level of *p* = 0.05, and a power of 1 − β = 0.95, a power analysis with G^∗^power (Faul et al., 2007) for the repeated measures ANOVA resulted in *N* = 53 to detect significant effects in the explicit as well as the implicit attitudes toward electric and gasoline cars. For this experiment, we used an affective priming paradigm. In the past, a small to moderate correlation between explicit and implicit measurements has been detected in priming paradigms (*r* = 0.21) (Cameron et al., 2012). Given *r* = 0.21, an alpha level of *p* = 0.05, and a power 1 − β = 0.95, a sample size of *N* = 241 is necessary to detect a relation between implicit and explicit measurements. The project has been made public before on osf^1^.

The experiment was conducted according to the ethical guidelines of the Helsinki Declaration and approved by the ethics board of the University of Regensburg (20-1740-101). We communicated all considerations necessary to assess the question of ethical legitimacy of the study. The question of data retrieving in the online experiment was coordinated with the data officer of the University of Regensburg. After study completion, the participants received a code to get course credit.

### Measurement

#### Demographic Questionnaire

Questions concerning sex, age, education state, frequency of practicing meditation, and the mean km driven by car in one week were asked, see Table 1.

**TABLE 1:** Demographic data and mean values (*SD*) of mindfulness and sustainability for car owners and non-car owners.

	**Car owner**	**Non-car owner**
Sex (% female)	63.22%	61.53%
Age	23.86 (6.12)	21.91 (2.82)
Degree		
High school	83.90%	93.60%
Master/Bachelor	14.95%	6.40%
Other	1.15%	
Km (car driving per week)	287.78 (1,177.37)	17.14 (30.50)
Frequency of meditation (min per week)	18.13 (96.99)	11.55 (35.27)
Importance of mobility	3.19 (0.85)	3.48 (0.55)
Sustainability	3.71 (0.84)	3.78 (0.74)
Mindfulness (FFMQ)	133.9 (17.01)	130.74 (17.43)
Future behavior	2.90 (1.07)	2.75 (1.02)
Past behavior	1.4 (0.64)	1.36 (0.64)
Meaning of sustainability for cars	5.22 (1.31)	5.31 (1.23)

#### Mindfulness Measurement

The FFMQ (Baer et al., 2008; Michalak et al., 2016) comprises 39 items with the five dimensions observing (“I notice the smell and aromas of things”), non-reactivity (“When I have distressing thoughts or image, I am able just to notice them without reacting”), acting with awareness (“When I do things, my mind wanders off and I’m easily distracted”), non-judging (“I think some of my emotions are bad or inappropriate and I shouldn’t feel them”), and describing (“My natural tendency is to put my experiences into words”). Each sub-dimension includes 7–8 items. Each item has to be rated on a five-point Likert scale from 1 = “applies very rarely” to 5 = “applies very often”. Cronbach’s alpha of the five sub-scales varied between 0.74 and 0.90 for the German version (Michalak et al., 2016). In the study presented here, Cronbach’s alpha of the five sub-scales varied between 0.78 and 0.89. The score for the subscales and the overall score were calculated by summing up the respective items per subject.

#### Sustainability and Mobility Measurements

##### Behavior

According to the studies of Hunecke and Richter (2019) and von Behren et al. (2018), (a) four questions (e.g., “The idea of sustainability is an important part in my life”) were asked with respect to the sustainability-related meaning in general (reliability measured with Cronbach’s alpha = 0.74), and (b) eight questions (e.g., “I am able to structure my daily life without a car”) regarding the importance of mobility in the own life (reliability measured with Cronbach’s alpha = 0.84). The participants had to answer on a five-point Likert scale from “does not apply” to “does apply.” For both scales, the mean has been calculated, see Table 1. Furthermore, three questions (e.g., “I am planning to buy an electric car in the future”) were asked regarding the future behavior (reliability measured with Cronbach’s alpha = 0.78), and two questions regarding the past behavior (e.g., “How often did you drive with an electric car in the past?”) with respect to electric mobility (reliability measured with Cronbach’s alpha = 0.43), which is not sufficient (Manca et al., 2019). The participants had to answer on a five-point Likert scale from “unlikely” to “very likely.” For all measurements, except the past behavior, the mean was calculated due to the good reliability. For the past behavior, only the answer to the question “How often did you drive with an electric car in the past” was considered for further analyses.

##### Meaning of the sustainability

With the use of semantic differential, the participants had to rate four to six features of the following two questions: “sustainability of cars is” (important–unimportant, of interest–not of interest, plays a role–plays no role, significant–insignificant) for me (reliability measured with Cronbach’s alpha = 0.90) and “electric cars are” (good–bad, appropriate–inappropriate, right–wrong, boring–funny, harmful–beneficial, and useful–useless) for me (reliability measured with Cronbach’s alpha = 0.88) (Manca et al., 2019). For each question, the mean has been calculated as well as the mean for both answers.

#### Explicit Evaluative Response

**FIGURE 1:**
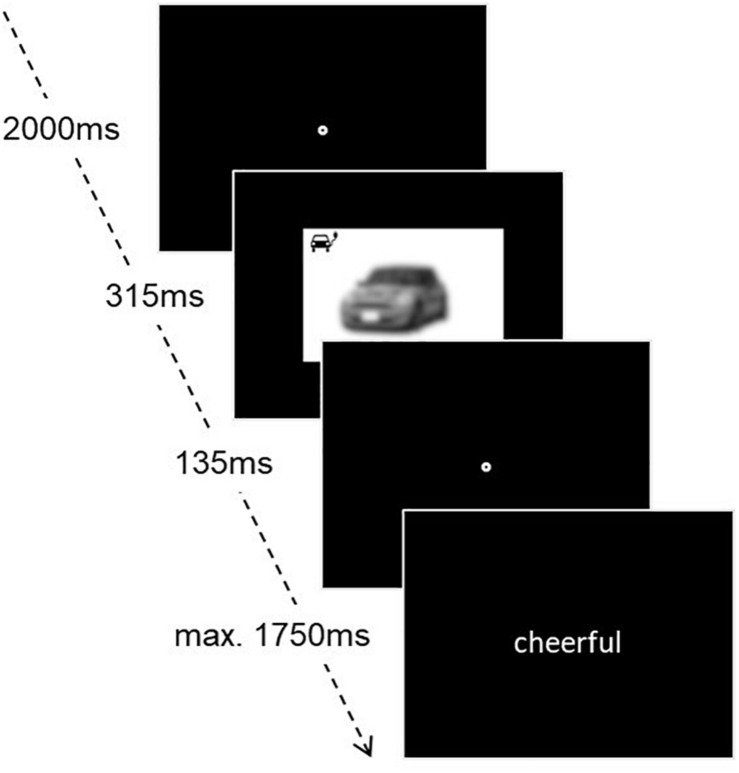
Experimental setup of the affective priming paradigm. The photo of the car has been blurred to grant the anonymity of the brand.

For the explicit rating task, pictures of 10 electric and 10 gasoline vehicles were used. The pictures show cars of different brands, with each electric car being matched to a gasoline car regarding color, attractiveness, and brand. The vehicle types cover a wide range of different vehicle classes (small cars, medium cars, luxury cars, and sport utility vehicles). However, to ensure that the electric cars are recognized as electric vehicles, an electric car sign was added in the left corner, see Figure 1. The explicit rating task consisted of three questions: (1) ATTITUDE: “what is your attitude toward the item on the screen?” (rating: “very negative”–“very positive”), (2) INTEREST: “how much are you interested in the item on the screen?” (rating: “not at all”–“very much”), and (3) APPEAL: “how appealing do you think is the item on the screen?” (rating: “not at all”–“very much”). The questions of the explicit ratings were adapted from the study of Hutcherson et al. (2008) that used questions on the attitude, the similarity, and the closeness they feel toward one person in a picture as explicit measurements (Hutcherson et al., 2008). All three questions were asked in a random order on each of the 20 pictures and rated on a seven-point Likert scale. The participants had 5 s to respond in order to provoke a spontaneous reaction. According to Hutcherson et al. (2008), two composite scores were created: first, the mean for each question for the electric and gasoline cars was separately calculated. After this, the means of the three questions were calculated.

#### Positive Affective Priming Task

To assess the implicit attitudes, an affective priming paradigm was used (Fazio et al., 1995; Hutcherson et al., 2008) with the same 20 pictures for electric and gasoline cars as in the explicit rating condition. Beforehand, a short practice trial was conducted with four pictures of neutral objects. Following an initial fixation point on the screen, which was shown for 2,000 ms, a picture of a car was presented briefly for 315 ms, followed by another 135 ms fixation point. Afterward, a word appeared on the screen, randomly picked from a list of 10 negative and 10 positive words, which were retrieved from the Berlin Affective Word List Reloaded (BAWL-R) (Võ et al., 2009). The participants had to indicate *via* the arrow keys if the word was positive or negative. They had to answer as quickly as possible, otherwise the word disappeared after 1,750 ms and the trial was repeated in the end, see Figure 1.

The participants made 7.2% mistakes on average, which were imputed using the mean of the reaction time for the respective picture. As in the study of Hutcherson et al. (2008), the difference between the reaction time of the negative and positive words of the same picture, separated for electric and gasoline cars, was used as indicator for the implicit attitude. For further statistical analysis, the mean value of electric cars and the mean value of gasoline cars were calculated. Accordingly, a higher difference score reflects a more positive evaluation.

### Procedure

The whole experiment was conducted online on the platform Jatos.org. It was programed using OpenSesame and SurveyJS, and it lasted about 15 min. The experiment was conducted for each participant in the following fixed order: in the beginning demographical data, the questions on mindfulness, sustainability, and future and past behaviors were surveyed. Subsequently, the explicit and the implicit tasks were conducted.

### Statistical Analysis

First, to test if there is a difference between the explicit rating of electric and gasoline cars, an ANOVA for the explicit composite scores was conducted with the within-subject factor “type of car” (electric, gasoline). Second, to test if there is a difference between the implicit affective ratings of electric and gasoline cars, an ANOVA with the difference scores between negative and positive words (reaction time) with the within factor “type of car” was calculated. Both ANOVAs were repeated with the covariates “ownership of cars” and “gender.” This was done in a second step, because the investigation of the influence of those factors was exploratory. Third, a correlation (Pearson correlation coefficient, only for degree Spearman correlation coefficient) between the demographic data (degree and age), mindfulness (FFMQ subscales) and sustainability in general, mobility, future and past behaviors, the meaning of sustainability for cars, and the explicit and implicit ratings for electric cars was calculated separately for the participants who own a car and those who do not own one. Based on these results in total, four regression analyses (method: Enter) were conducted, two for car owners (explicit attitudes vs. implicit attitudes) and two for non-car owners (explicit attitudes vs. implicit attitudes).

## Results

### Explicit Evaluative Responses

Regarding the composite score, a significant main effect of “type of cars,” *F*(1, 164) = 37.65, *p* < 0.001, η*_*p*_*^2^ = 0.187, appeared. Electric cars (*M* = 3.88, *SD* = 1.02) had a higher explicit composite score than gasoline cars (*M* = 3.43, *SD* = 0.91). If the “ownership of car” was integrated as a covariate in the analysis, the interaction between “type of cars” and “ownership of cars” did not reach significance (*p* = 0.062). In addition, the factor “gender” has no influence on the results, if it was integrated in a further analysis.

### Implicit Evaluative Responses

Regarding the reaction time difference score, there was no main effect of “type of cars,” *F*(1, 164) = 0.538, *p* = 0.464, η*_*p*_*^2^ = 0.003. If the “ownership of car” was integrated as a covariate in the analysis, the interaction between “type of cars” and “ownership of cars” was not significant (*p* = 0.880). In addition, the factor “gender” has no influence on the results, if it was integrated in a further analysis.

### Correlations and Predictions of Explicit and Implicit Evaluative Responses

Pearson and Spearman correlations between the implicit and explicit evaluations of e-cars and age, degree (Spearman correlation), mindfulness, sustainability in general, the importance of mobility in life, and the km driven by car in a week were calculated separately for the participants who own a car and those who do not own one.

For the participants who own a car, there were significant correlations for the rating of electric cars and sustainability in general (*r* = 0.29, *p* = 0.006), future behavior (*r* = 0.41, *p* < 0.001), age (*r* = −0.24, *p* = 0.026), and degree (*r*_*s*_ = −0.23, *p* = 0.030): the explicit rating was higher if the participants rate themselves more sustainable and want to use an electric car in the future, for younger people and the ones with a high school degree compared with the ones with a higher degree. The implicit rating of electric cars and the overall score of the FFMQ (*r* = 0.26, *p* = 0.014) and its dimension non-reactivity (*r* = 0.22, *p* = 0.04) showed that more mindful participants and those who have a higher non-reactivity rate electric cars more positive in the affective priming paradigm. Based on these correlation results, two regression analyses were conducted for the participants who own a car. For the explicit rating of electric cars, we included age, degree, future behavior, and sustainability in general as predictors, for the implicit rating, the overall score of the FFMQ, and its dimension non-reactivity. The overall regression model for the explicit rating explained 24.4% of the variance [corrected *R*^2^ = 0.207, *F*(4, 81) = 6.55, *p* < 0.001]. The only predictor that reached significance was future behavior, *b* = 0.30, *p* = 0.003. The implicit rating of e-cars for the car owners could not be significantly predicted by the overall score of the FFMQ, *b* = 1.20, *p* = 0.150 and its dimension non-reactivity, *b* = 1.53, *p* = 0.602 [*R*^2^ = 0.072, corrected *R*^2^ = 0.05, *F*(2, 84) = 3.27, *p* = 0.043].

For the participants who do not own a car, there were two positive correlations between the explicit rating of electric cars and future behavior (*r* = 0.52, *p* < 0.001) and the mindfulness dimension describing (*r* = 0.25, *p* = 0.028): the explicit rating was higher if the participants are able to express their feelings. Besides, there was one negative correlation between the implicit rating of e-cars and the mindfulness dimension acting with awareness (*r* = −0.27, *p* = 0.016). The regression for the explicit rating explained 29.7% of the variance [corrected *R*^2^ = 0.278, *F*(2, 75) = 15.85, *p* < 0.001]. Only the predictor future behavior, *b* = 0.514, *p* < 0.001, reached significance. The overall regression model for the implicit rating explained 7.4% of the variance [corrected *R*^2^ = 0.062, *F*(1, 76) = 6.06, *p* = 0.016]. The predictor dimension of the FFMQ acting with awareness was significant, *b* = −5.86, *p* = 0.016.

### Relation of Explicit and Implicit Evaluative Responses

There was neither a correlation for the explicit and implicit evaluative responses for the electric cars (*r* = −0.043, *p* = 0.708) nor for the gasoline cars (*r* = 0.012, *p* = 0.915).

## Discussion

The main results of this study demonstrated a difference in the explicit and implicit affective evaluations of electric and gasoline cars: in the explicit rating, electric cars are rated more positively than gasoline cars. There was no difference in the implicit affective rating. In addition, explicit and implicit evaluations of electric and gasoline cars did not correlate. Furthermore, the correlations of the investigated factors mindfulness, future and past behaviors, sustainability in general, and the implicit and explicit ratings depended on car ownership.

The investigation of the affective evaluations of sustainable environmental behavior is important because affective motives were identified as relevant factors in environmental psychology (Steg, 2005). However, our results demonstrate that electro-mobility is only explicitly rated as more positive. There is no difference in the affective implicit judgment of electric and gasoline cars. One explanation for this might be the social desirability bias: the pressure to be a social responsible human being in times of climate change may lead the participants in our study to express explicitly a positive attitude. Within the affective priming paradigm, the preference for e-mobility could not be demonstrated, and both measurements do no correlate. This incongruence of attitudes toward sustainability is also known as “green-washing” or symbolic social responsibility in contrast to authentic responsibility (Steiner et al., 2018). While “green-washing” has more or less been described on the group level, the results of the study presented here show the green-washing effect on the individual level (Donia and Tetrault Sirsly, 2016). The green-washing effect regarding general sustainability has already been investigated by Steiner et al. (2018). In their study, 114 executives from different companies participated in an explicit rating and implicit measurement. In the explicit task, they had to rate the importance of various sustainability aspects in the five categories of economical, ecological, social, institutional, and cultural sustainability. Implicit attitudes were measured with the Standard IAT with the categories “sustainable” and “unsustainable” and “good” and “bad” (Steiner et al., 2018). The use of the different implicit measurements might contribute to the different results in their study compared with our study. On the other hand, the IAT focuses on the more or less cognitive implicit concept with the help of the affective priming paradigm (Brand and Ekkekakis, 2018). Another reason for the different results might be that in the study of Steiner et al. (2018), the participants had to perform an implicit rating task regarding the sustainability toward more broader concepts, such as re-use, economic sustainability, and social commitment. Those concepts are more abstract than the implicit rating of gasoline and e-cars, and the participants might not be as involved in those questions. Furthermore, and even though in both, explicit and implicit measurements, a positive rating could be shown in their study, the correlation between both concepts was close to zero, which is in line with the data we obtained in the study presented here. However, implicit measurements are not the better or the more accurate measurement; both explicit and implicit measurements should be used as equally valuable methods. Accordingly, a more holistic comprehension of attitudes toward specific aspects of sustainability is possible, which is important for the change toward sustainable behavior. The combined use of explicit and implicit measurements can help to reveal underlying attitudes and explain the expressed intention in the future to pay for sustainable goods, as in this case, e-cars.

A second interesting result of our study is that the two attitudes toward e-cars are correlated and predicted by different factors, also dependent on car ownership. While the explicit rating of the electric cars correlates with sustainability in general, future behavior, age, and degree, the implicit rating is correlated with the overall mindfulness score of the FFMQ and the subscale of mindfulness “non-reactivity” for the car owners. For the non-car owners, future behavior, as well as the mindfulness dimension of “describing,” is correlated with the affective explicit rating of the e-cars. The implicit rating of e-cars correlated negatively with the mindfulness dimension “acting with awareness.” This gives a hint that both types of attitudes are related to different concepts and might be altered by different training programs. Regarding the explicit rating of electric cars, our data could not demonstrate a predictive effect of the past behavior, whereas it is assumed that habits and past behaviors can predict pro-environmental intentions (Klöckner and Verplanken, 2018). The missing effect might be due to the low use of electric cars in the past. However, if people have higher values of sustainability in general and if they plan to use electric cars more often in the future, they rate electric cars explicitly as more positive. Only the future behavior was a significant predictor. Until now, it is assumed that positive emotional images can increase the likelihood of pro-environmental behavior (Hine and Gifford, 1991). Our study gives a hint that one aspect of pro-environmental behavior, the meaning of sustainability in general, is related to positive emotional images of electric mobility at least in car owners, even though a predictive effect could not be found in our data.

However, one has to be careful suggesting such an influence due to the correlational nature of the results in this study. In our study, the implicit evaluation of electric cars is positively correlated with mindfulness in general and especially the dimension non-reactivity (car owners), whereas it is negatively correlated with acting with awareness (non-car owners). Sustainability in general is not correlated to the implicit rating, providing evidence for the dual pathway model of attitudes, which comprises a more “conscious” and a more “unconscious” awareness route (Cameron et al., 2012). Non-reactivity might have a regulatory function, because impulsive behavior is suppressed, and the awareness is directed toward personal norms (Hunecke and Richter, 2019). The effect of mindfulness on implicit affective ratings of sustainable behavior has not been studied until now. Only in the study of Hutcherson et al. (2008) it was shown that even after just a few minutes of loving-kindness meditation, the feelings of social connection and positivity toward novel individuals on both implicit and explicit tasks (the same as used in this task) were ameliorated. Crescentini et al. (2014) showed that 8 weeks of mindfulness orientated meditation increased the implicit attitude toward religious self-representations in people who had a low existing religious affiliation before. However, mindfulness is a broad term, which needs to be better conceptualized (van Dam et al., 2018) to investigate the causal relevance for implicit affective responses. Different forms of meditations can be used as elements of mindfulness interventions as, for example, attention-based, constructive, and deconstructive meditation practices (Dahl et al., 2015).

A further result in this study is that the relation between varying psychological and demographical factors and explicit and implicit affective ratings differs depending on car ownership. These results indicate that the relevance of the own life circumstances should be integrated in research when investigating sustainable attitudes toward different questions. Similar conclusions were drawn by Richter and Hunecke (2020), who found that an important prerequisite for behavioral change is forming the intention to change one’s behavior in a predecisional stage, which is closely intertwined with explicit attitudes and mindfulness. However, none of the participants in this study owned an e-car, and most of the participants were students who cannot afford the purchase of an e-car. Consequently, it could be speculated that the participants without a car were not even in a predecisional stage, since the topic is irrelevant to them. Even though they might explicitly appreciate e-mobility, they implicitly know that they are not able to possess one in the near future, which might influence their ratings and the connections to other psychological variables. Accordingly, it would be interesting to investigate these relations in a sample with car owners.

### Limitations

Several possible limitations have to be discussed. First of all, the study was implemented as an online study. Despite the fact that we did not perform a correlation to a laboratory-based study, several effects in the area of cognitive psychology have been successfully replicated in online experiments (Kochari, 2019), indicating that the online procedure used here is acceptable. However, the affective priming paradigm is only one possible implicit measurement among others and has a high affective component. It would also be interesting to investigate more cognitive implicit measures, such as the IAT (Brand and Ekkekakis, 2018). The dispositional mindfulness measurement used in this study has a high attention-based focus. For this, it might be worth to include a dispositional mindfulness measurement in further studies, which integrates the dispositional measurement of loving awareness as one aspect of the constructive form of mindfulness (Dahl et al., 2015). Besides, the participants of this study were mainly students. The experiment should be repeated with the participants who are more the target group who might be interested in purchasing an e-car. In addition, a correlation between implicit and explicit measurements could not be carved out. One reason for this might be that only 168 participants could be included in the analysis of the experiment. Another reason might be that the explicit and implicit measurements are not perfectly comparable because they are based on different metrics. Furthermore, it was assumed that electric cars in general are perceived as more sustainable options over regular cars even though this is heavily dependent on the sources of electricity that is used in order to charge the electric car. In addition, the attitudes toward hybrid cars were not investigated in this study, though it is well known that they are rated more positive than gasoline cars. The participant’s knowledge, perception, and attitude toward this are something that would have to be included in future studies to gain a full picture of the sustainability behavior and meaning.

## Conclusion

In summary, we demonstrated different explicit and implicit attitudes toward e-cars and gasoline cars. Electrical cars are only explicitly rated as more positive than gasoline cars. On the one hand, this result might contribute to the green-washing debate in social groups; on the other hand, it might be regarded as just two sides of the coin, which has to be investigated further due to the factors that might influence not only sustainable attitudes but also sustainable behavior.

## Data Availability Statement

The dataset presented in this study can be found in the following online repository: https://osf.io/sk2tp/.

## Ethics Statement

The studies involving human participants were reviewed and approved by Ethics Committee of the University of Regensburg (20-1740-101). The participants provided their written informed consent to participate in this study.

## Author Contributions

PJ designed and supervised the study, developed the theoretical framework, performed the analyses, and wrote the first draft of the manuscript. FS and PH helped planning and conducting the experiment and processed the experimental data. RR conceived the presented idea and developed the theoretical framework. All authors discussed the manuscript.

## Conflict of Interest

The authors declare that the research was conducted in the absence of any commercial or financial relationships that could be construed as a potential conflict of interest.

## Footnotes

1 https://osf.io/sk2tp/
